# The probiotic bacteria and their encapsulated forms as food components: Survival, effects and quality

**DOI:** 10.3934/microbiol.2026001

**Published:** 2026-02-10

**Authors:** Elena Nikitina, Dmitrii Khrundin

**Affiliations:** Department of Meat and Milk Technology, Institute of Food Technology and Biotechnology, Kazan National Research Technological University, 420015, Kazan, Russian Federation

**Keywords:** probiotic, encapsulation, functional meat, milk, plant foods, viability, stability

## Abstract

In this review, we summarised the information on probiotic bacteria and their encapsulated forms as functional food components, emphasising survival, technological effects, and product quality. Probiotics from genera such as *Bifidobacterium* and *Lactobacillus* are widely used to prevent and manage gastrointestinal and systemic disorders. However, their efficacy is often limited by the loss of viability that occurs during processing, storage, and passage through the gastrointestinal tract. In this article, we analyse the major encapsulation techniques (spray drying, freeze drying, emulsification, extrusion, coacervation, and electrospraying/electrospinning) and emphasise the important function of encapsulating materials, such as proteins, polysaccharides (e.g., alginate, pectin, and chitosan), lipids, and their combinations. Particular focus is given to mixed polymer systems and co-encapsulation with cryo- and protective agents, which can enhance resistance to acid and bile, increase survival by 1–2 log units, and help maintain bioactive compounds. We also consider fermented dairy products, cheese, meat products, and plant-based matrices as carriers for free and encapsulated probiotics. Thus, we show that, when properly selected, these systems can improve microbial stability, modulate proteolysis and lipolysis, and enhance antioxidant and antimicrobial properties without compromising sensory quality. Particular emphasis is placed on emerging plant-based beverages and alternative substrates that could enable probiotics to be consumed by lactose-intolerant and vegetarian populations. Overall, we present encapsulation as a promising strategy for designing next-generation functional foods with predictable probiotic survival and tailored technological and sensory characteristics.

## 1. Introduction

Probiotics are specific strains of live microbial cells. The Food and Agriculture Organisation (FAO)/World Health Organisation (WHO) defines them as “live microorganisms which, when administered in adequate amounts, confer a health benefit on the host” [1]. Food is usually the source of probiotics for humans, but they can also be obtained from pharmaceutical preparations. These formulas include mono- or mixed cultures of live microbial cells. Only a narrow range of species among the diversity of microorganisms have probiotic properties. These mostly include strains from the genera *Bifidobacterium* spp. and *Lactobacillus* spp. [2], including *Bifidobacterium animalis subsp. lactis* [3],[4], *Lactobacillus acidophilus* [5], *Lacticaseibacillus rhamnosus* [6], *Lactiplantibacillus plantarum* [7],[8], *Lacticaseibacillus paracasei* [7], and *Limosilactobacillus reuteri* [8],[9].

**Figure 1.**
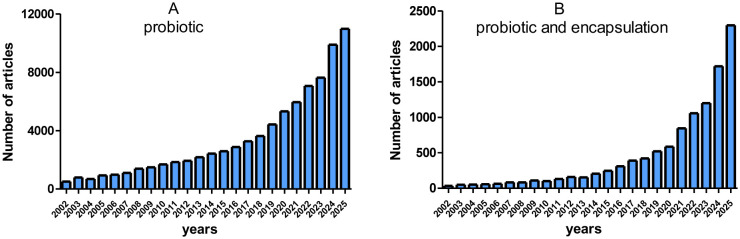
Trends in the number of articles on probiotics, as well as probiotics and encapsulation.

As noted in a joint report by the WHO and the FAO, in order for the definition of probiotics to be met and for benefits to be guaranteed to the person taking them, six criteria that define these bacteria as probiotics must be met by the formula [10]: 1) Resistance in an acidic gastric conditions; 2) optimal survival in bile acid conditions; 3) high adhesion to intestinal mucosa and/or epithelial cells; 4) an antimicrobial effect on potentially pathogenic intestinal bacteria; 5) the ability to limit the adhesion of pathogenic microorganisms to the intestinal mucosa; and 6) the ability to dissolve bile acid salts. The scientific community's interest in probiotics is illustrated by the increasing number of scientific articles published between 2002 and September 2025 (analysis of the number of articles from the website www.sciencedirect.com using the search term ‘probiotic’) (Figure 1A).

The use of probiotics is a common strategy for the prevention and treatment of several diseases, such as inflammatory bowel disease, irritable bowel syndrome (IBS), atopic skin diseases, acute infectious diarrhoea, and antibiotic-associated diarrhoea [11]–[16]. Even when probiotic strains meet the established criteria, their performance in real food products is frequently compromised by process- and storage-related stresses, so that fewer viable cells ultimately survive passage through the gastrointestinal tract.

Probiotic properties are mainly analysed *in vitro* at the screening stage, and bacterial strains demonstrate high probiotic potential. However, when tested *in vivo*, the effectiveness of these same strains often decreases sharply. Encapsulating probiotics can help maintain their effectiveness when they are used in living organisms or stored in food or pharmaceutical products. A search for ‘probiotic and encapsulation’ on ScienceDirect.com, based on data on the number of scientific publications for the period 2002–2024, shows a growing interest in the field of encapsulation among the scientific community (Figure 1B). Overall, the trend shows an increasing number of articles as encapsulation is considered a key technology for improving the stability, viability, and functionality of bacteria in application areas.

Microencapsulation technology can enhance the delivery of probiotics by shielding them from environmental and physiological factors, such as pH, oxygen and temperature, while ensuring they reach the colon. The survival of encapsulated probiotic bacteria is significantly impacted by encapsulating materials. This review article provides a comprehensive overview of materials used for the microencapsulation of probiotic bacteria, offering a thorough comparison of all food encapsulation materials.

In this review, we focus on the use of probiotics and their encapsulation in fermented food products of various origins. There are quite a few reviews by leading scientists devoted to encapsulation methods [17]–[21]. This field is expanding quickly, including the development of encapsulating probiotic cells with protective agents, also known as co-encapsulation [22]–[24]. Although health is benefited by probiotics, their use is limited due to the reduction in the viability of probiotic cells during processing, storage, and delivery to the gastrointestinal tract [25].

Our focus of this review article is to take a closer look at contemporary research on the impact of encapsulating probiotic bacteria in food technology. Our aim is to identify how this technological approach affects the complex properties of food products and bacteria.

The market for encapsulated food ingredients is experiencing growth due to increasing demand for functional products, natural additives, and convenient forms of consumption. However, manufacturers face challenges relating to strict regulatory compliance and product quality control. The juridical aspect of encapsulation in food products is regulated by international and national standards, which are aimed at ensuring product safety, quality, and transparency. The major requirements relate to ethics, risk assessment, technology approval, labelling, and compliance control (Table 1).

Methodologically, this review involved searching for and selecting scientific articles based on a structured search of the ScienceDirect, PubMed, and Web of Science databases using the keywords ‘probiotic’, ‘encapsulation’, ‘microencapsulation’, ‘functional foods’, ‘dairy products’, ‘meat’, and ‘plant products’. Original research articles and reviews published between January 2002 and September 2025 were considered, with preference given to peer-reviewed, quantitative, English-language studies containing data: Encapsulation, microencapsulation, probiotics, survival, food systems (fermented milk products, cheese, fermented meat and sausages, plant products), *in vitro*/*in vivo* assessments of survival and functionality, physicochemical, and sensory properties of food. Regulatory aspects covered EFSA, FDA, and EAEU requirements, as well as GMP/GLP standards. Studies not related to food applications, lacking information on bacterial survival, or focusing exclusively on non-probiotic microorganisms were excluded.

**Table 1.** Legal regulation of food ingredient encapsulation across countries and regions.

Region/Country	Key Regulatory Documents/Agencies	Key Requirements and Regulatory Features
International level	*Codex Alimentarius*	Guidelines on food safety: Risk assessment methods and technological controls. Standards are not mandatory but are frequently used as a basis for national regulations.
	Hazard Analysis and Critical Control Points system (HACCP)	The HACCP system requires monitoring of production stages where safety risks may arise.
USA	Food and Drug Administration (FDA)	New food ingredients/additives must demonstrate safety. Manufacturers may submit applications to the FDA for approval to use such ingredients. Mandatory labeling requirements: disclosure of product composition and potential allergens. Regulation depends on the status of ingredients (food additives or new dietary ingredients).
EU	1. Regulation No 178/2002 («On laying down the general principles and requirements of food law, establishing the European Food Safety Authority, and laying down procedures in matters of food safety»). 2. Regulation No 1333/2008 («On food additives»). 3. Regulation No 1935/2004 («On materials and articles intended to come into contact with food»). European Food Safety Authority (EFSA)	General principles of food safety across all stages of the production chain. Food additives must be approved and included in the EU's unified list, with specified conditions of use (requires safety assessment and confirmation of technological necessity). Requirements for packaging materials: migration of substances from encapsulating materials into the product must not affect its safety, composition, smell, or taste.
Russia	1. Technical Regulation of the Eurasian Customs Union (TR CU 021/2011) «On Food Safety». 2. Federal Law No. 52 «On Sanitary and Epidemiological Welfare of the Population»	Food safety requirements extend to manufacturing, storage, transportation, sale, and disposal of all food products, including items obtained using encapsulation technologies. These products must be safe for human health and must not mislead consumers. The introduction of new food products, additives, and production technologies is permitted only when they meet established sanitary and epidemiological standards.
Japan	Food Sanitation Act	Regulation covers food products, additives (including natural flavorings), equipment, and packaging that comes into contact with food.

## 2. General encapsulation techniques

Researchers are extremely interested in techniques for encapsulating microbial cells. In general, those working in this field distinguish the following methods: Emulsification, spray drying, freeze drying, extrusion, the coacervation process, electrospraying [26]–[28], and methods of bionanotechnology [29]–[33].

Emulsification is one of the most common methods of co-encapsulating probiotics. This process involves two phases: A dispersed phase containing a cell suspension in a polymer and a continuous phase consisting of an oil (vegetable or mineral) or organic solvent, to which a surfactant is also added [34].

Spray drying is a well-known microencapsulation method that has been used in the food industry since the late 1950s to prevent the oxidative degradation of flavouring oils by converting them from a liquid to a powder. However, this method has limitations when it comes to encapsulating probiotics, as high temperatures can negatively affect cell stability and preservation. To improve the preservation of the membrane and bacterial proteins, carbohydrates are used in the technology: Sorbitol, xylose, mannitol, sucrose, lactose, maltose, glucose, dextrose, inulin, maltodextrin, galactooligosaccharide, potato starch, and fructooligosaccharide [35].

Freeze drying, or lyophilisation, is also a widely used and convenient method for extending the shelf life of microencapsulated probiotics. This technique involves preliminary encapsulation of cells in the presence of cryoprotectants [21],[36],[37].

Extrusion can also be used for microencapsulation. During extrusion, vibration is caused by the passage of a colloidal solution containing cells through a nozzle, and at the same time, a vibration frequency is applied to form a laminar jet [38].

The coacervation process is a technique that involves the formation of a coacervate. This is the result of the phase separation of biopolymers at a specific composition, temperature, or pH of the solution. Ultimately, microparticles are formed by the precipitation of the coacervate around the major ingredients [39]. The strength of resulting probiotic microspheres can be increased by adding cross-linking agents, either enzymatic or chemical [40].

Electrospraying and electrospinning technologies were first developed in the 1930s. However, they have only recently been applied to the encapsulation of probiotics [41],[42]. Electrospraying involves obtaining micro- and nanoscale droplets from a liquid solution through the combined action of electricity and spraying. A similar mechanism is used for electrospinning to produce micro- or nanofibres.

Among the array of methodologies employed, emulsification and extrusion stand as the most economical and uncomplicated. While these methods are compatible with food products, the low long-term stability of emulsions and wide particle size distribution make scaling and quality control difficult. Although spray and freeze drying are well-established industrial methods, they subject cells to thermal or osmotic stress and require rigorous optimisation of carrier composition and process parameters to maintain viability, a more complex approach that increases the cost of the technology. Despite encapsulation technologies being seemingly well known, interest in these methods continues unabated. Despite significant progress, encapsulation technologies for live probiotic cells remain far from fully standardised, and there are open questions regarding how process parameters and wall materials jointly shape long-term stability and *in vivo* performance. Therefore, in future work, researchers should focus not only on protecting cells from processing stresses, but also on understanding how different encapsulation strategies interact with specific food matrices and gastrointestinal conditions to deliver consistent probiotic functionality.

## 3. Encapsulation as a strategy to improve the viability of probiotics

Probiotics can be added to products in a variety of ways. The simplest method is to add a pure, viable culture to a food product and then mix and ferment it. When storing the product, it is important to consider its metabolic characteristics: Lactic acid produced by lactobacilli can lead to excessive acidification.

Encapsulation is an effective method of obtaining pure or tableted forms of probiotics, and is therefore used in the pharmaceutical industry. Probiotics are obtained as a pharmaceutical preparation using spray drying or freeze-drying technologies. When free cells are used, the average survival rate of probiotics after drying is 70–85%. Cell survival can be increased by encapsulating the cells on carriers using bioactive compounds, which range from low-molecular-weight compounds such as cyclodextrin and maltodextrin to complex polymers with a high molecular weight. These include modified starches, proteins, gums, and lipids [23],[43]–[47]. In addition to encapsulation, freeze drying technology requires the use of a cryoprotectant. A number of prebiotics can act as cryoprotectants [48], poly-γ-glutamic acid [49], sucrose, inulin [50],[51], powdered milk, soy polysaccharides, trehalose [52], and others. The use of microencapsulation in combination with cryoprotectants in freeze-drying increases the survival rate of probiotic bacteria by around 25%, compared to drying without protective additives.

However, this expensive method is not suitable for producing functional foods. Encapsulating microorganisms is a potential way to increase the survival of probiotics added to different food products [53]–[56].

### 3.1. Encapsulation materials

Natural biopolymers (proteins, polysaccharides, lipids, etc.) and synthetic biopolymers are widely used as components for encapsulating probiotics [57]–[62]. The use of polymers suitable for encapsulation is described below and presented in Table 2.

Gelatin is a protein and peptides obtained by hydrolysis of collagen (from bones, skin, and connective tissues of animals). It dissolves easily in hot water and forms a gel when cooled; it is also compatible with polar solvents. Its biodegradability, biocompatibility, and non-immunogenicity in physiological environments make it a promising material for encapsulation and delivery of probiotics [59],[60]. Moreover, the addition of inulin and galactooligosaccharide on the survival of microencapsulated probiotics in alginate beads coated with chitosan in a simulated digestive system, yogurt, and fruit juice was tested [19],[63],[64]. However, gelatin's animal origin also limits its use, due to its odor and that it is prohibited for vegans, for example.

The advantages of polysaccharides include high stability, low immunogenicity, and high availability. Alginate hydrogels are well-known for being an economical and versatile method of encapsulating probiotics. These consist of two monosaccharide units, D-mannuronic acid (M) and α-L-guluronic acid (G), which are linked together in a 1–4 configuration. The gels are insoluble in acidic environments, which can protect probiotics in such conditions. The carboxyl group of alginates can cross-link with divalent cations to form a hydrogel [57].

Chitosan is a positively charged polysaccharide made up of D-glucosamine and N-acetylglucosamine units joined together by β(1→4) bonds. It is produced commercially by partially deacetylating chitin extracted from crustaceans. Due to its inhibitory effect on microorganisms (including lactic acid bacteria), chitosan is typically used as a coating or shell rather than a capsule [65].

**Table 2.** Polymers that encapsulate probiotics.

Bacteria Systems	Polymer	Effect	Survival, CFU/mL or EE, %*	Reference
*Bifidobacterium and L. acidophilus*	Polymerized whey protein	Well-received sensory property and stable storage properties.	Population remained 10^6^ CFU/mL during storage.	[53]
*Lactobacillus acidophilus 5 and Lacticaseibacillus paracasei 01*	Galactooligosaccharides and inulin	Prebiotics reach the intestine in high amounts and improve human health through modulation of the gut microbiome.	Population remained 10^7^ CFU/mL during storage.	[66]
*Bacillus licheniformis*	Alginate microparticles	Alginate particles exert a protective effect on bacteria, maintaining their viability in the shrimp intestine.	EE 51.3 %	[67]
*Bifidobacteria and Lactobacillus*	Chitosan	Protecting probiotics from environmental influences, increasing their stability and ensuring effective delivery to the intestine.	Data not available	[68]
*Lacticaseibacillus rhamnosus GG*	Sodium Alginate and arabinoxylan composite	Encapsulated probiotics showed higher viability than free probiotics in simulated gastrointestinal conditions.	Data not available	[69]
*Lactobacillus acidophilus*	Sodium alginate, whey protein isolate, kappa-carrageenan, and citrus pectin	The highest encapsulation efficiency was observed for whey protein isolate, followed by K-carrageenan and citrus pectin.	EE 45.6 %	[54]
*Lactobacillus acidophilus*	Pectin, whey protein	Microcapsules of pectin with multi-layered layers of whey protein concentrate contribute to greater protection and viability of probiotics.	EE 62.6–87.9 %	[70]
*Lactiplantibacillus plantarum*	Pectin, pea protein isolate	These emulsions possess the capacity to encapsulate probiotics and provide highly effective protection for pro-biotic viability during *in vitro* simulated gastrointestinal digestion.	EE 52.65 %	[71]

* Survival CFU/mL—Remaining probiotic population at the end of shelf life; EE—Encapsulation efficiency.

A number of researchers are investigating the possibility of using chitosan and its synergistic combinations with other materials [72], sodium alginate, and arabinoxylan [73],[74] for microencapsulation of probiotics.

In recent years, pectins have been suggested as a new, widely applicable material for encapsulation. This is because pectins are versatile and inexpensive, can be adapted to requirements, and are beneficial to health [75],[76]. Research is underway into developing nanofibres based on apple pectin using the electrospinning method, which exhibits good barrier properties [77]. Pectin microcapsules containing *Lactobacillus acidophilus* have been obtained through external ion gelation, followed by the adsorption of whey protein and pectin to form multilayer shells [70]. Probiotic films have been produced by incorporating four probiotics (*Lacticaseibacillus casei casei*, *Bifidobacterium bifidum*, *Lactobacillus acidophilus*, and *Lacticaseibacillus rhamnosus*) into citrus pectin gels [78]. The possibility of combining pectin with other biopolymers is also being investigated. Fucoxanthin, for example, is a biologically active compound with antitumour, antioxidant, antimicrobial, and anti-inflammatory properties, but it is unstable, which creates problems in product development, especially with regard to shelf life. The synthesis of nano-chitosan-pectin and the encapsulation of isolated fucoxanthin by ion gelation has been proposed [79]. A double emulsion using pea protein isolate and pectin has been developed for encapsulating *Lactiplantibacillus plantarum* [71].

Thus, in hydrogels based on alginate and pectin, ionic cross-linking of carboxyl groups with divalent cations creates structures that are comparable to “egg cartons”. These structures are dense and limit the diffusion of protons, oxygen, and bile salts towards the cells. Protein and polysaccharide aggregates provide additional buffering and hydrophobic protection. This protection helps shield cell membranes from acidic and surface-active food components [57].

An analysis of recent years' results has revealed the major advantages and disadvantages of materials used for probiotic encapsulation (Table 3), as well as some contradictions in the associated research.

**Table 3.** Polymers that encapsulate probiotics.

Polymer	Advantages	Disadvantages	Applications
Sodium alginate	Cost-effectiveness; resistance to the acidic environment of the stomach; ability to form hydrogels.	Limited biocompatibility; risk of capsule aggregation when the density difference with the forming solution is small.	Widely used for probiotic microencapsulation.
Chitosan	Positively charged polysaccha-ride; exhibits inhibitory action against microorganisms; used as a coating or shell.	May inhibit the growth of some probiotics, so it is often used in combination with other polymers.	Used in combination with alginate (alginate-chitosan capsules) to enhance the protection of probiotics.
Pectin	Availability; adaptability to specific requirements; compatibility with prebiotics.	Depends on the source of origin; requires optimization of gelation conditions.	Applied for microencapsula-tion of probiotics and for creating probiotic films.
Gelatin	Availability; ability to form various shapes (granules, plates, membranes); presence of functional groups.	Animal origin limits use (specific odour; not suitable for vegans); gelation depends on temperature.	Used in combination with other polymers (e.g., pectin) for microencapsulation.

1) Effectiveness of probiotic protection. Different studies show discrepancies in data on the survival of probiotics after microencapsulation. The choice of encapsulation method and material should depend on how sensitive a particular strain is to stress factors such as acid, temperature, and enzymes. Using synergistic combinations (e.g., alginate + chitosan or pectin + gelatin) can improve probiotic protection and capsule properties.

For example, some studies show that alginate-chitosan capsules provide high protection efficiency, while others indicate a risk of decreased viability of the bacteria within the capsules compared to the initial material.

2) Colonisation resistance. Even when probiotics are successfully delivered to the intestine, they are not always able to colonise the mucous membrane due to competition with commensal microbiota. Studies in mice indicate that animals with normal microflora exhibit lower probiotic colonisation than germ-free animals. It is necessary to evaluate not only the survival of probiotics in the gastrointestinal tract, but also their ability to colonise the intestine and their therapeutic efficacy.

3) Standardisation of methods. The lack of uniform standards for probiotic production and evaluation methods makes it difficult to compare the results of different studies. For instance, researchers may employ distinct probiotic strains, experimental conditions, and efficacy evaluation criteria. The requirements for describing probiotic products (e.g., indicating strains, the number of CFUs, and storage conditions) should be adhered to.

4) Clinical efficacy. Despite numerous studies, data on the therapeutic efficacy of probiotics remains inconclusive. Certain studies have indicated advantageous outcomes in particular circumstances (e.g., antibiotic-associated diarrhoea), while others have failed to demonstrate any substantial effect.

5) Process scaling. Laboratory microencapsulation methods are not always scalable to an industrial level due to the difficulty of controlling parameters such as capsule size uniformity and property stability. Industrial production requires specialised encapsulators to automate the process and ensure consistent results. It is imperative that microencapsulation methodologies be incorporated into extant foodstuffs or pharmaceuticals manufacturing facilities.

## 4. Encapsulation for products of animal origin

Microencapsulation is actively used in food technologies that involve fermentation, such as the production of fermented milk products, cheeses, dry-cured sausages, and meat. Undoubtedly, one of the key tasks of encapsulation is to increase the preservation of bacteria, especially probiotic bacteria, during product storage and digestion in the gastrointestinal tract. On the other hand, the use of encapsulation techniques should not impair the quality of food products or reduce their technological and sensory properties.

### 4.1. Encapsulation for dairy products and cheese

The encapsulation of probiotics in yoghurt and other fermented milk products is considered an effective way to increase cell survival during production, storage, and passage through the gastrointestinal tract [80]. However, probiotic cultures (e.g., *Lactobacillus* and *Bifidobacterium*) are exposed to an acidic environment, cooling, oxygen, and mechanical stress during manufacturing and storage, which often reduces their number to below 10⁶–10⁷ CFU/g by the end of their shelf life. This level is insufficient for a pronounced effect [81].

Microencapsulation creates a physical barrier, usually made of alginate, starch, milk proteins, or gums, which protects cells from acid, oxygen, and storage stress, thereby increasing their survival in yoghurt and simulated gastrointestinal conditions. From a technological point of view, hydrocolloid capsules are the most convenient and effective option, including those based on a combination of materials such as sodium alginate, alginate-starch, and alginate-inulin [80]. This widespread approach effectively protects probiotic cells in yoghurt, increasing their survival by one to two orders of magnitude during storage and in simulated gastrointestinal conditions [82]. In encapsulated form, probiotics maintain higher concentrations (≥10⁷–10⁸ CFU/g) throughout the storage period and experience a slower decline in numbers than free cells [83]. Similar data were obtained for the Doogh drink containing encapsulated *L. acidophilus* and *L. rhamnosus* in chitosan. There was a significant improvement in organoleptic scores [84].

The presence of protein matrices (casein, whey proteins formed during fermentation) must be taken into account when talking about fermented milk products and probiotics. This food matrix is highly compatible with polysaccharides, such as starches and mucilage [85],[86]. Using plant polysaccharides as a basis for cell encapsulation improves capsule distribution in milk gel and can stabilize it further through protein-polysaccharide networks. Yogurt with encapsulated *L. acidophilus* was more aromatic; its flavor scores after 5 weeks of storage were 7.67, whereas the non-encapsulated variant scored only 7.13. This aligns with prior probiotic studies where encapsulation protects flavor compounds during extended storage [87]. Combined systems (e.g. alginate + starch, alginate + gum, protein + maltodextrin) often have a synergistic effect, expressed as increased encapsulation efficiency and improved lactic acid bacterial viability and product texture. Yoghurts with encapsulated probiotics (whey protein alginate was used) maintain their viscosity during storage because the encapsulating materials stabilise the yoghurt structure [86],[89].

Given the high number of lactic acid bacteria in yoghurt when encapsulated cells are used, a high degree of acid accumulation can be assumed. However, encapsulation reduces the metabolic activity of cells, so yoghurt acidifies more slowly, which improves pH stability during storage, reduces post-acidification processes, and improves organoleptic properties [81]. With the right capsule size (usually <1 mm), the structure of the yoghurt is practically unaffected; a slight ‘grittiness’ is possible, which can be compensated for by selecting the right matrix and fillers [87].

An increasing number of studies have demonstrated the potential of using cheese as a carrier for probiotic microorganisms. For instance, commercial probiotics such as *L. acidophilus*, *Bifidobacterium* spp., *Lc. casei*, *Lc. paracasei*, and *Lc. rhamnosus* can supplement the primary starter culture in Cheddar cheese [88],[90] or protect against pathogenic bacteria [91]. However, the issue of how to deliver probiotics to the gastrointestinal tract and preserve them during the ripening and storage of cheese remains.

Modern research shows that encapsulation is a successful strategy for increasing the survival rate of probiotics in cheese. Using co-encapsulated *Lp. plantarum* in synbiotic cheese reduces its decline, increases antioxidant activity, reduces proteolysis intensity, and produces a more stable cheese matrix [92]. After 35 days of ripening, the count of probiotic *B*. *bifidum* cells encapsulated in alginate and κ-carrageenan was 10 times higher than that of free cells. After 35 days of ripening, the population of encapsulated probiotic bacteria in the cheese decreased by 1.03 log CFU/g, while in the variant with non-encapsulated cells, it decreased by 2.60 log CFU/g [88]. Furthermore, the encapsulated forms demonstrated superior survival in the aggressive simulated conditions of the gastrointestinal tract, which confirms the protective and stabilising role of hydrogel matrices [59].

Pecorino cheese was produced using encapsulated *L*. *acidophilus*, *B*. *longum*, and *B*. *lactis* [93]. Higher proteolytic activity was observed due to the presence of probiotic bacteria released from alginate granules. In the case of cheddar cheese, the variant with encapsulated *B. bifidum* cells showed a pH level 0.3–0.5 units higher, and protein content and moisture 0.5–1% higher than the non-encapsulated variant [88]. Using microencapsulated *Lc*. *paracasei* cells via enzymatic gelation in feta cheese slowed primary proteolysis and titratable acidity accumulation rates compared to samples containing free cells. Conversely, the addition of *Lc. paracasei* significantly increased the antioxidant activity of ultrafiltration feta cheese. Furthermore, the composition of the microcapsule shell had a significant impact on the cheese's textural characteristics, particularly its hardness and ‘stickiness’ (stringiness). In this case, the hardness of Feta cheese in the variant with encapsulated cells was twice that of the control [94]. In contrast, the survival of microencapsulated probiotic *Lc*. *paracasei* in low-fat mozzarella cheese was practically no different from the control variant with free cells [95]. This does not refute the positive results obtained by other authors, but highlights the importance of carefully selecting the type of capsule, microorganism strain, and cheese variety for the use of microencapsulated cells.

In the case of milk protein-based food products, it is important to note that alginate-protein capsules in milk gels form a multi-scale matrix by incorporating into the casein and whey protein network through hydrogen bonds and electrostatic interactions. This matrix buffers the local pH and reduces the direct impact of lactic acid on the membranes of probiotic bacteria. Additionally, the hydrophobic regions of milk proteins offer partial protection to cells against surface-active lipids and aromatic components. This complex structural integration partly explains why encapsulated cells often exhibit slower acidification kinetics and higher viability than free cells in dairy products such as yoghurt and cheese [96].

Therefore, the encapsulation of probiotics in milk-derived protein matrices is a promising and technologically feasible technique for the dairy industry, as it enables stability of cultures to be combined with controlled changes in product structure.

### 4.2. Meat product

The use of starter cultures in meat products can be a convenient way to introduce probiotics into the human body. Lactic acid bacteria play a key role in preparing dried meat products using this technology. They undoubtedly ensure the product's microbiological safety through the synthesis of organic acids (mainly lactic acid) and bacteriocins [97],[98]. From a sensory point of view, they also contribute to the characteristic flavour, aroma, texture, and colour of the finished product. Different strains of LAB affect the meat matrix in different ways, enabling the targeted selection of starter and probiotic cultures to produce product characteristics.

*Lp*. *plantarum* is often used in the production of salami, chorizo, and sujuk sausages. *Lp. plantarum* strains contribute to the sour taste and stable red colour of these products by reducing nitrite levels and forming stable nitroso pigments [99]. Furthermore, the intensive formation of acid contributes to the denaturation of muscle proteins, leading to the compaction of minced meat and the formation of the desired dense consistency [100]. A moderate amount of acids is synthesised by *Latilactobacillus sakei*, which prevents excessive acidity and enables complex flavour nuances associated with proteolysis and lipolysis to unfold. The formation of a balanced aroma is helped by this effect on raw meat materials [101]. *Pediococcus* can impart light, sweet notes due to its metabolism of pentoses. The addition of *Pediococcus* to mixed starter cultures was found to significantly increase total amino acid content, including those responsible for sausage flavour [102]. Taste, aroma, and texture can all be influenced by a wide range of lactic acid bacteria: *Lactococcus lactis*, *Leuconostoc citreum*, *Lacticaseibacillus paracasei*, and *Limosilactobacillus reuteri* [103].

An increasing number of studies suggest that meat products can have functional properties, including the use of probiotic strains of lactic acid bacteria in starter cultures [104]–[107]. The growing interest in using meat products to carry probiotics raises the question of how to protect probiotic cells from adverse factors. Encapsulation is also a convenient strategy in this case. For example, the use of encapsulated *Lp*. *plantarum* cells in chorizo sausages resulted in a higher bacterial count than the free cell variant, without compromising quality [108]. In the same study, the authors observed more intense moisture loss in the variant with encapsulated cells after 20 days of chorizo sausages ripening, accompanied by an increase in fat percentage. From a metabolic perspective, encapsulation slowed acid accumulation, with the pH in the variant with encapsulated *Lp. plantarum* being 0.5 units lower of control. In another study, microcapsulated *Lp*. *plantarum* was added to Milano-type salami to create a probiotic product [109]. The probiotic Milano-type salami did not differ from the control in terms of sensory characteristics, but it contained a higher number of lactic acid bacteria.

Using microencapsulated probiotic cells of the species *Lm*. *reuteri* and *B*. *longum* as co-cultures in meat starter cultures of the species *Pediococcus pentosaceus* and *Staphylococcus carnosus* enhances the bactericidal effect against *Escherichia coli* [110] due to increased probiotic survival. Studies of *Lm*. *reuteri*, whose cells were immobilised in chitosan-coated alginate microcapsules [111] or in alginate beads prepared with different mucilages/gums [112], have evidenced the enhanced antimicrobial effect against food-borne pathogens.

In terms of their physical and chemical properties, dry sausages or meat are characterised by their high ionic strength (due to the presence of NaCl), as well as the presence of nitrites/nitrates, reactive carbonyl compounds, and lipoic acid products. Additionally, polysaccharide and protein coatings on capsules reduce cell contact with these reactive substances by acting as diffusion barriers and partially binding metal ions that catalyse oxidation. In the presence of proteins and hydrocolloids, the coating retains moisture around the cells and reduces water activity locally, thereby stabilising the membrane lipids and proteins of probiotic cells. Therefore, we can conclude that, in fermented meat, matrices rich in polysaccharides and proteins that encapsulate probiotic cells limit their direct contact with salts, reactive compounds derived from nitrites and lipid peroxidation products during the ripening process. These matrices also bind metal ions involved in oxidative reactions, thereby reducing membrane damage and preserving cell viability [113].

Thus, fermented meat products and sausages enriched with probiotic strains of lactic acid bacteria are a promising way of delivering probiotics to the human body. Starter cultures ensure microbiological safety by synthesising acids and bacteriocins, and they form the desired sensory characteristics, including taste, aroma, texture, and colour. Encapsulating probiotics enhances their survival in the unfavourable conditions of the meat matrix while preserving the product's organoleptic properties and enhancing its antimicrobial effect against pathogens. Thus, combining probiotic starters with encapsulation technology creates new opportunities for developing innovative, effective functional meat products.

## 5. Encapsulation for plant product

Some population groups, including those with milk protein allergies or lactose intolerance and strict vegetarians, cannot consume dairy products [53],[66],[67],[114],[115]. Therefore, there is a need to offer consumers an alternative to fermented milk products, which can be achieved by studying new non-dairy ingredients and products as probiotic carriers. Cereals, legumes, fruits, vegetables, and other plant crops are among the many options that should be highlighted [54],[68],[116]–[118]. These foods contain a large amount of nutrients and biologically active substances absent from animal milk, such as antioxidants, bioflavonoids, glucans, and polysaccharides. This is why plant-based fermented products are considered a new source of probiotics and are the focus of research and development around the world [56],[69],[114],[119]–[123].

There is a genuine interest in developing functional beverages containing non-dairy probiotics as they offer a healthy alternative to dairy probiotics and are suitable for consumers with lactose intolerance or who wish to avoid cholesterol. Phytochemicals, such as phenolic compounds [124]–[126], and specifically, avenanthramides andavenacosides A and B [127], are considered beneficial to human health. They reduce the risk of degenerative diseases, malignant neoplasms, and inflammatory processes by lowering oxidative stress and inhibiting macromolecular oxidation.

The use of fruit raw materials is proposed [128]–[130]. The influence of traditional lactic acid bacteria cultures, such as *Str*. *thermophilus* and *Lactobacillus delbrueckii subsp*. *bulgaricus*, on the quality characteristics of fermented milk, combined plant-based and oat drinks [123], and whey [131],[132] using *Lactobacillus acidophilus*, is being studied. The role of national and new beverages as reservoirs of probiotic lactic acid bacteria is emphasised in a number of studies examining their diversity. These include yoghurt-like national beverages such as borhani and lavan, as well as fermented beverages based on date palm juice [133]–[135], buckwheat [136], millet [137], rice, oats and barley, carob, and tiger nuts [135],[138],[139].

There is a growing trend towards using fruit raw materials [128]–[130]. The influence of traditional yoghurt cultures containing lactic acid bacteria (*Streptococcus thermophilus* and *Lactobacillus delbrueckii subsp*. *bulgaricus*) on the quality characteristics of fermented milk and plant-based oat beverages is being studied [55].

The survival rate of the probiotic *Bacillus coagulans GBI-30 6086* was assessed in pectin fillers containing jussara and passion fruit pulp [140]; the potential of the *Lactiplantibacillus plantarum FNCC-0461* strain, which was obtained from the traditional Indonesian product ‘dadih’, was studied; and it was proposed that the strains *Lactiplantibacillus plantarum BR9*, *Lactiplantibacillus plantarum P35*, and *Lactobacillus acidophilus IBB801*, as well as two substrates (5% wheat bran and 10% red beet/carrot), could form the basis of a new beverage [55],[141]. The microbiological profile and consumer properties of each product are specific (Table 4).

**Table 4.** Alternative probiotic plant-based products.

Probiotic	Product	Characteristic	Survival, CFU/mL	Reference
*Bifidobacterium longum*, *B. infantisand*, *B. breve*, *Streptococcus thermophilus*, *Lactobacillus delbrueckii subsp. bulgaricus*	Yoghurt-like fermented smoothie beverage	Smoothie beverages maintained the probiotic counts required for the health claim.	Population remained 10^7^ CFU/mL during storage.	[128]
*Lactiplantibacillus plantarum*	Fermented fruit and vegetable beverages	Increase in the total phenolics and total flavonoids content. Increase of the antioxidant activity of the fermented beverages.	Data not available	[129]
*Lactiplantibacillus plantarum 299v*, *Lactobacillus acidophilus La-5*	Fermented dairy-oat beverage	Improving the stability of the product and its consumer properties.	Population remained 10^6^ CFU/mL during storage.	[130]
*Streptococcus thermophilus*, *Lactobacillus delbrueckii subsp. bulgaricus*, *Lactobacillus acidophilus La-5*, *Bifidobacterium animalis subsp. lactis BB-12*	Fermented buckwheat beverage	Milk allergen free gluten free fermented cereal beverages respond to celiac disease and food intolerance needs.	Population remained 10^7^ CFU/mL	[136]
*Limosilactobacillus fermentum LAB-1*, *Levilactobacillus brevis LAB-5*	Borhani – yoghurt-like fermented product	Nutritional products designed to support digestive well-being.	Data not available	[133]
*Consortia of LAB*	Fermented oat lactose-free sauce	The sauce can be used by people with lactose intolerance and vegetarians.	Population remained 10^6^ CFU/mL during storage.	[139]
*Lactiplantibacillus plantarum subsp. plantarum B1-6*, *Leuconostoc mesenteroides subsp. mesenteroides DSM 20343*	Fermented legume-based beverages	By reducing antinutritional factors and increasing phenolic content, protein digestibility, and viscosity, fermentation significantly upgrades the nutritional value and functional characteristics of the final product.	Data not available	[142]
*Lactiplantibacillus plantarum 299v*, *Weissella confusa 2LABPT05*	Fermented finger millet product	The product has antidiabetic potential and bifidogenic effects, and consequently its consumption might positively impact blood glucose levels and the human gut microbiota.	Population remained 10^8^ CFU/mL	[137]
*Consortia of LAB*	Plant-based beverages (tiger nut, rice, carob)	The beverages showed good sensory attributes, floral notes, balanced aroma profile and pleasant sensory qualities.	Population remained >10^8^ CFU/mL	[135]

Encapsulated probiotic bacteria have potential applications in plant product technology. In the case of plant products, encapsulation increases the survival rate of probiotic bacteria and preserves biologically active plant components. For instance, the encapsulation of *Lc*. *rhamnosus GG* in calcium alginate matrices containing hydrocolloids (carrageenan, agar, and gelatin) resulted in an increased survival rate and stabilisation of total phenolic content in fermented black goji berry drinks [143]. Microencapsulation of *Levilactobacillus brevis* and *L*. *delbrueckii subsp*. *lactis* increased resistance in a simulated gastric environment and a simulated intestinal environment, as well as in bile, compared to non-encapsulated forms [144]. Another study entailed using a symbiotic culture in combination with prebiotics to produce oat yoghurt. This showed increased resistance to gastric juice, as well as more intensive enrichment with four essential amino acids (glycine, valine, leucine, and glutamine) and eight non-essential amino acids (alanine, serine, proline, asparagine, thioproline, aspartic acid, glutamic acid, and α-aminopimelic acid) [145]. During storage, fermented tomato juice (both control and juice with encapsulated *Lp. plantarum*) had the lowest lycopene content, while *L. delbrueckii* had the highest. Samples containing the encapsulated bacterial mixture exhibited increased turbidity, stability, and antioxidant activity compared to the control sample, which remained the most transparent [146]. Encapsulated probiotics (*L*. *acidophilus* and *B*. *bifidum*) in pasteurised grape juice retained ten times more cells after 60 days of storage than the variant with free cells [147]. Furthermore, the high preservation of the encapsulated lactic acid bacteria may reduce the concentration of patulin in juices, as demonstrated in fermented apple juice containing encapsulated, cross-linked keratin-chitosan hydrogel with *Lc*. *rhamnosus* [148].

Thus, plant-based foods such as grains, fruits, vegetables, and legumes (e.g., oats, buckwheat, palm juice, dates, and tiger nuts) are a promising alternative to dairy products for delivering probiotics, particularly for those with lactose intolerance, milk protein allergies, or who follow a vegetarian diet. Fermenting these plant matrices using strains of *Lp*. *plantarum*, *Str*. *thermophilus*, *Bifidobacterium* spp., and other lactic acid bacteria not only preserves the probiotics' viability, but also increases their phenolic content, antioxidant activity, and sensory properties. The encapsulation of probiotics in alginates, chitosan, and hydrocolloids has been demonstrated to enhance their survival during storage and within the gastrointestinal tract. Furthermore, this process stabilises bioactive components and reduces the content of toxins, such as patulin. Therefore, combining plant substrates with encapsulated probiotics paves the way for innovative functional beverages and products that have proven health benefits [149]–[151].

It is well known that plant products contain a variety of polyphenolic compounds and bioflavonoids, which are particularly prevalent in colored fruits and berries. These substances have a bacteriostatic effect to some extent, reducing the survival rate of lactic acid bacteria. In fruit- and cereal-based beverages, alginate-pectin capsules form non-covalent complexes with phenolic compounds via hydrogen bonds and hydrophobic interactions. This mitigates the potential pro-oxidant and membrane-active effects of certain polyphenols on probiotics while stabilising these phytochemicals and reducing their bacteriostatic effect. It should be noted that including prebiotics in the capsule shell serves as a bacterial substrate and modifies the matrix's hydration and gelation, thereby increasing its stability during storage [152].

In the future, there will be an expectation of the expansion of the raw material base by the inclusion of exotic fruits, rare grains, and root vegetables, as well as the development of multilayer and nanocapsule systems with controlled release. A key area of focus will be the development of synbiotic compositions (probiotics and prebiotics) to promote intestinal colonisation and enrich products with essential nutrients. Furthermore, there are several problems that need to be solved if this is to be achieved. These include how to increase production without increasing the cost of encapsulation, how to standardise methods for controlling the viability of probiotics, and how to confirm the preventive potential of such products through long-term clinical trials.

## 6. Conclusion

Encapsulation of probiotics is generally considered a key technology for improving their survival and functionality in dairy, meat, and plant-based food matrices. This approach compensates for the *in vivo* reduction in strain effectiveness caused by loss of viability during processing, storage, and passage through the gastrointestinal tract. Combining spray and freeze drying, emulsification, extrusion, coacervation, and electrochemical methods with protective agents increases probiotic survival and enables therapeutic levels to be achieved in products. The degree of cell protection, the stability of bioactive components, and the texture of the product are determined by biopolymers (alginate, pectins, proteins, starches, chitosan, and their compositions). In dairy, meat, and plant-based systems, capsules based on combined matrices have been shown to preserve probiotics, improve organoleptic properties, and enhance antioxidant and antimicrobial activity. In summary, the effect of encapsulation on probiotic cells in food systems of different origins (plant or animal) is mediated not only by physical protection, but also by chemical interactions, such as ionic cross-linking, acid buffering, modulation of oxygen and bile salt diffusion, binding of reactive species and phenolic compounds, and restructuring of the distribution of water and dissolved substances at the capsule-matrix interface. Understanding these mechanisms is important not only for the empirical improvement of survival, but also for the rational selection of materials for walls and food matrices.

The rational selection of strains, encapsulating materials, and the type of food matrix enables the design of functional products with predictable probiotic survival and controlled sensory and nutritional characteristics. There are promising areas of research, such as co-encapsulation with prebiotics and phytoactive components, as well as the study of the effects of encapsulated forms in clinical trials.

## Use of AI tools declaration

The authors declare they have not used Artificial Intelligence (AI) tools in the creation of this article.
